# Digital health initiatives can take better cognizance of marginalized communities in India

**DOI:** 10.7189/jogh.12.03069

**Published:** 2022-10-21

**Authors:** Jatin Khaimani, Angad P Singh Bhatia, Arnav Jeurkar, Dipti Rao, Narendra Chirmule, Pallavi Misra, Rajashree Kadam, Santhiya Karuppieh, Shruthy Seshadrinathan, Smritie Sheth

**Affiliations:** 1 Nalagenetics Pte Ltd., Singapore; 2 Punjab Engineering College (Deemed to be University), Chandigarh, India; 3 Walmart Global Tech, Dallas, Texas, USA; 4 Art X Company, Bangalore, India; 5 SymphonyTech Biologics, Philadelphia, Pennsylvania; 6 Shoolini University, Kasauli, India; 7 Gautam Buddha University, Greater Noida India; 8 ImmunitoAI, Bengaluru, India; 9 Institute of Chemical Technology, Mumbai, India

Who are we leaving behind as we move further into a digital world? In the information age, digital technology is ubiquitous in nearly all aspects of human life. Can we truly walk together into the future? In the 1900s Mead posited that a healed femur is the first sign of civilization. In that vein, health is at the foundation of all human pursuit. Where do the ubiquitous and fundamental meet? “Digital health” lies at the intersection.

This study examines digital health – accessing health care services digitally – in three aspects: its potential in India, extant public health infrastructure, and its need in marginalized communities. The study explores the major challenges in the field and offers recommendations for conceptual solutions to professionals working in digital health, students, physicians, and policy makers.

“Digital health” is the use of information and communication technologies in medicine and other health professions to manage illnesses and health risks and to promote wellness [1]. Amongst other digital technologies, mobile phone technology would form the base of a Maslowian pyramid, a theory of motivation which states that five categories of human needs dictate an individual's behaviour. The demand for mobile phone technology as reported by the Indian Cellular and Electronics Association (ICEA), states that people in rural regions spend almost the same percentage of their household budget (25%) on mobile phones as urban residents (26%) [2]. Significantly, the Pradhan Mantri Grameen Digital Saksharta Abhiyan (PMGDISHA), the Indian government’s largest rural digital access program, states on its website their aim to make at least one person in every family digitally literate, with a goal to train 60 million rural resident adults by 2020.

By 2022, 60% of India will use mobile phones and place the country second only to China in the penetration of mobile phone technology [2]. While the anecdotal trends suggest the spread of digital technology typically occur in urban centres, India's numbers tell a different story. ICEA reports that rural India observed a 35% per year rise in the number of internet users in 2018 while urban regions showed 7% growth – all primarily facilitated by mobile phones [2]. In rural India, smartphone penetration has risen from 9% in 2015 to 25% in 2018 [2]. Mobile applications and software enable wide access to telemedicine. To describe, review and understand the multi-factorial processes involved in digital health, we have categorized the process flow into three connected categories among providers and patients. These categories are: aware to engage, test to treat, and order to ship, illustrated in **Figure 1**.

**Figure 1:**
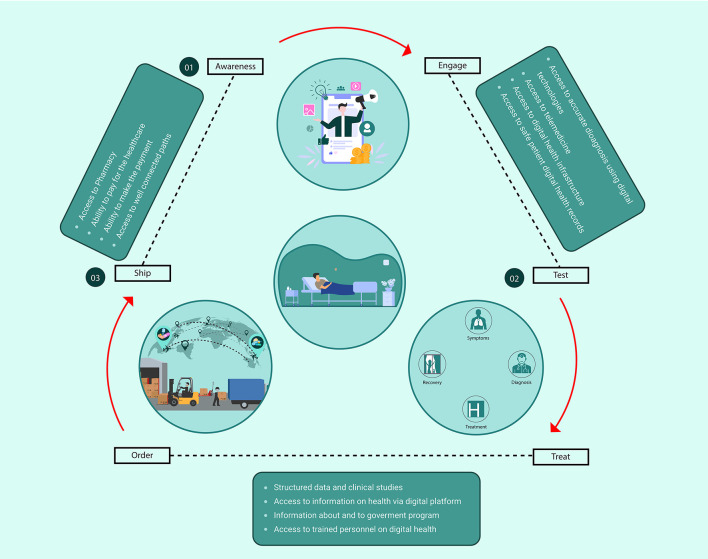
Potential of digital health to health care seekers and providers. In order to describe, review and understand the multi-factorial processes involved in digital health, we have classified into three categories namely, from aware to engage, from test to treat, and from order to ship. Each category has touchpoints which provide or enable health care to the two “functions” of each category: patient and provider respectively. The arrows in the model describe the flow between these functions and its touchpoints – the red solid arrows indicate journeys fulfilled by the provider, while the black dotted arrows represent the user-enabled journey. Moreover, the blockers in each category are listed alongside the red solid arrows.

## THE POTENTIAL OF DIGITAL HEALTH IN INDIA

McKinsey reports that the investments in digital health-based enterprises have soared since 2019 [3]. Some core components of the digital health ecosystem recognised by the Food and Drug Administration (FDA) include e-health (electronic health), m-health (mobile health), health 2.0 devices, telemedicine, telehealth, public health surveillance, precision medicine, health tracking strategies, self-tracking and sensor technologies, and medical imaging and information systems. Mobile applications and artificial intelligence/machine learning-based technologies can support clinical decisions, improve diagnostic accuracy, and provide safe and effective treatment options of “non-serious” conditions. The overall benefits of data-driven digital health include: i) reduced inefficiencies, ii) improved access, iii) reduced costs, iv) increased accuracy, and v) feasibility of administering personalized medicines.

**Figure:**
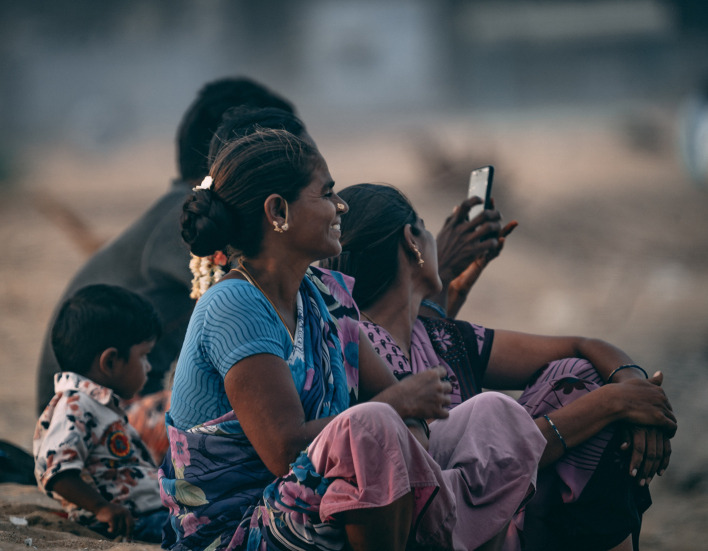
Photo: “Aware to Engage, Test to Treat, and Order to Ship”, India’s health care sector urgently requires research into the adoption challenges of digital health amongst its most vulnerable citizenry as evidenced by the digital divide highlighted in the pandemic. Source: https://unsplash.com/photos/CBucCjSOuuY.

In 2019, the World Telecommunication/ICT Indicators Database reported that only 41% of India had access to the internet [4]. Those lacking internet access tend to identify as poor people, live in remote or rural areas, the elderly, women, and people with disabilities [5]. Our viewpoint seeks to identify the requirements of digital health technologies with the potential to transform access to health care for these marginalized communities in India.

## THE STATE OF DIGITAL HEALTH FOR MARGINALIZED COMMUNITIES

Marginalized communities are socially excluded and confined to the periphery of society. These groups of people are often denied participation in political, cultural, and social activities. Excluded from mainstream society, they are often severely and chronically handicapped as a community, their capability to contribute to society, reduced. They are typically identified as women, people with disabilities, scheduled castes (Dalit), scheduled tribes, elderly or aged people, children, and sexual minorities [5]. These communities comprise the vulnerable, oppressed, underrepresented, and undercounted populations – those who have been deprived of their basic needs [5.] They may have some money but no access to basic health. The health status of the marginalized communities in India has been reported as deficient on the socio-economic grounds, and a summary of these reports is shown in Table 1 of the article [6]. Further, marginalization can change over time; new groups can appear. This exploratory paper focuses on the digital health challenges faced by communities marginalized based on i) physical disability, ii) tribal origins or identities, and iii) economic challenges through a review of available literature and interviews with key opinion leaders involved in the Indian health care system. These challenges are important to recognise as the country adopts various digital health infrastructures like Ayushman Bharat Digital Health Mission (ABDHM) by the National Health Authority (NHA). **Figure 1** highlights areas of attention required in each of the three categories of digital health.

## HOW THE PUBLIC HEALTH INFRASTRUCTURE IN INDIA CURRENTLY SERVES ITS POPULATION

The health care system in India is organized into three levels: sub-centres and primary health centres (PHCs) administer primary care, community health Centres and sub-district hospitals offer secondary care, while tertiary care is provided by medical colleges and district or general hospitals, all providing free or nearly-free service. In rural areas, PHCs are the first stop for treatment, mandated to be set up within 8 km of any residential community in the country. Doctors here are supported by Accredited Social Health Activist (ASHA) workers who often belong to the local community that they serve.

In 2017, an analysis of health care in South Asian countries found that in India, financial risk protection stood at 17.9%, while coverage for prevention and treatment for select conditions stood at 83.5% [7]. The following year, the ABDHM program was launched with the aim to provide universal health coverage to India’s citizens under two linked schemes: health and wellness centres and the National Health Protection Scheme. The first, is to provide free diagnostic services and medication for primary care, and the second is to protect vulnerable citizens against financial risk arising from hospitalization. A recent innovation of the program is the Unified Health Interface, an open network modelled on the now ubiquitous Unified Payments Interface (UPI), that is designed to enable interoperable digital health service delivery that can service rural areas as well by providing longitudinal access to patients [8].

## THE IMPACT OF COVID-19 ON THE DIGITAL HEALTH INDUSTRY

Global lockdowns across 2020 and 2021 created a particular spike in demand for remote medical consultation and sales of online medical supplies. In April 2020 the Economic Times in India reported that major e-pharmacies in the country experienced a nearly 100% rise in demand for supplies as the average consumer avoided store visits. Inequalities in access to such support were highlighted in this time, caused by a lack of knowledge about health systems, poverty compounded by a lack of digital currency, low digital literacy skills, and related issues. UI/UX design rarely addresses challenges faced by marginalized communities in using their platforms; it is locally active non-governmental organisations (NGOs) and community services in rural communities that provide support to these marginalized communities by offering mobile data connections and educating locals about Arogya Setu (disease tracking), CoWIN (vaccination access and booking), and E-Sanjeevini OPD (a telemedicine initiative). Marginalized communities in wealthy urban areas grappled with predominantly English language interfaces. The Supreme Court had to intervene with a Suo Moto cognizance in May 2021, to direct CoWIN and the Arogya Setu application to be made available in regional languages [9]. Although COVID-19 was devastating for many marginalized communities, it was instrumental in providing a fillip to India’s slow-moving digital health industry, further boosted by initiatives like ABDHM and the National Digital Health Mission. India’s health care sector urgently requires research into the adoption challenges of digital health amongst its most vulnerable citizens. Technological training in health care needs to begin early. Globally, while public health care is looking to move beyond electronic health records (her) to a more holistic knowledge-driven care model, in India, despite major leaps ahead in technological interventions, the system struggles to provide equitable access to health care, as also found by our interviews with key opinion leaders [10].

## THE WAY FORWARD

Digital literacy rates have climbed since the COVID-19 pandemic. Digital interventions in health care can help close the gap in digital access amongst marginalized communities. The key opportunities to overcome these access challenges lie in enabling last-mile access via ASHA workers (awareness to engage), the training of rural doctors to deliver digital health solutions (test to treat) and incentivizing privately held supply chain solutions to service marginalized communities (order to ship).
